# Electrophysiological correlates of temporal numerosity adaptation

**DOI:** 10.3389/fnins.2024.1349540

**Published:** 2024-03-05

**Authors:** Paolo A. Grasso, Irene Petrizzo, Francesca Coniglio, Roberto Arrighi

**Affiliations:** ^1^Department of Neuroscience, Psychology, Pharmacology and Child Health, University of Florence, Florence, Tuscany, Italy; ^2^Department of Physics and Astronomy, University of Florence, Florence, Italy

**Keywords:** numerosity adaptation, EEG, temporal numerosity, steady-state visual evoked potential, numerosity perception

## Abstract

**Introduction:**

Much research has revealed the human visual system is capable to estimate numerical quantities, rapidly and reliably, in both the spatial and the temporal domain. This ability is highly susceptible to short-term plastic phenomena related to previous exposure to visual numerical information (i.e., adaptation). However, while determinants of spatial numerosity adaptation have been widely investigated, little is known about the neural underpinnings of short-term plastic phenomena related to the encoding of temporal numerical information. In the present study we investigated the electrophysiological correlates of temporal numerosity adaptation.

**Methods:**

Participants were asked to estimate the numerosity of a test sequence of flashes after being exposed to either a high or low numerous adapting sequence. Behavioral results confirmed the expected underestimation of test stimulus when this was preceded by a high numerous sequence as compared to when preceded by a low numerous sequence.

**Results:**

Electrophysiological data revealed that this behavior was tightly linked to the amplitude of the steady-state visual evoked (ssVEP) response elicited by the test stimulus. When preceded by a high numerous sequence, the test stimulus elicited larger ssVEP responses as compared to when preceded by a low numerous sequence with this pattern being robustly correlated with behavior. Finally, topographical maps showed that this difference was mostly evident across two antero-posterior distributed clusters of electrodes and correlated with changes in functional connectivity.

**Discussion:**

Taken together, our results suggest that visual plastic phenomena related to the encoding of temporal numerosity information reflect changes in rhythmic evoked activity that are likely related to long range communications between distinct brain regions.

## Introduction

Both human and non-human species possess the ability to rapidly estimate numerical quantities with relatively good accuracy (Dehaene, 2011). This ability, known as numerosity perception, is thought to be subserved by the recruitment of a specific mechanism, the abstract numerosity system, activated during the enumeration of elements in a scene.

Numerosity perception has been extensively studied in both its spatial and temporal counterparts. Spatial numerosity refers to the ability of estimating a series of spatially segregated items presented at the same time while temporal numerosity refers to the ability of estimating temporal sequences, such as flashes or sounds, presented within the same spatial location. Although psychophysical evidence showed that the two modalities are somehow intertwined (Barth et al., 2003; Arrighi et al., 2014), other studies report that the encoding of spatial and temporal numerosity could be subserved by independent systems. For instance, Cavdaroglu and Knops used fMRI to show that parietal cortex is involved in the extraction of spatial numerosity, while the encoding of temporal numerosity would be mostly subserved by temporal regions (Cavdaroglu and Knops, 2019). Other evidence come from studies on children where it was found that formal math abilities correlate positively with sensitivity for estimation of spatial but not temporal numerosity (Anobile et al., 2018).

One characteristic that spatial and temporal numerosity perception share is plasticity which make both these mechanisms highly susceptible to adaptation (Burr and Ross, 2008; Arrighi et al., 2014; Aagten-Murphy and Burr, 2016; Grasso et al., 2021a,b, 2022a). For example, visually inspecting for a few seconds a large number of items scattered on a given region of space results in the perceived numerosity of a subsequent ensemble presented in the same area to be strongly underestimated with the opposite occurring as a consequence of adaptation to low numerosity. Likewise its spatial counterpart, temporal numerosity can get adapted as the exposure to sequences of numerous stimuli (i.e., sequence of flashes) induces an underestimation of the subsequent test sequence while exposure to a low numerous sequence induces an overestimation (Arrighi et al., 2014). This phenomenon is robust and has been replicated also within the auditory domain (Grasso et al., 2022a) and in cross modal conditions including touch (Togoli and Arrighi, 2021). However, while determinants of spatial numerosity adaptation has been widely explored in both their physiological correlates (Castaldi et al., 2016; Grasso et al., 2022b) and the relationship with non-numerical exogenous factors (Grasso et al., 2021a,b, 2022a), temporal numerosity adaptation has received much less attention. However, a deeper understanding of the characteristics of plastic phenomena associated with the perception of temporal numerosity would allow to shed light on whether the two numerical modalities are independent processes or rather reflects different branches of a unique system involving distributed brain regions. To explore this point, in the current study we exploited the high temporal resolution of EEG to investigate the electrophysiological correlates of temporal numerosity adaptation.

## Materials and methods

### Participants

Sample size was determined through a power analysis conducted using G*Power 3 Software (Faul et al., 2007). The analysis indicated that a total sample of 16 participants would be needed to detect medium effects (*f* = 0.30) with 95% power and an alpha level of 0.05. Eighteen participants took part in the study. Two participants were excluded because of outlier behavioral performances (i.e., greater than three scaled median absolute deviation). The final sample then comprised sixteen participants (mean age: 25.38 years, SD: 7.08 years; 7 males) with normal or corrected to normal visual acuity and no diagnosis of dyscalculia. All participants provided written informed consent before participation.

The research was approved by the local ethics committee (“*Commissione per l’Etica della Ricerca,”* University of Florence, 23rd September 2021, n. 174).

### Apparatus and stimuli

The whole experiment was conducted in a dimly lit and sound attenuated room with participants seated 51 cm away from a LED monitor (20 inches, refresh rate: 60 Hz, 1920 x 1,080-pixel resolution) with their head placed on a chinrest.

Stimuli consisted of black discs (diameter: 3° degrees) briefly flashed (40 ms) on a gray uniform background at an eccentricity of 8.5° to the right of a central fixation point. Adapting stimuli consisted of either 64 (High numerosity adaptation) or 16 (Low numerosity adaptation) flashes presented along an 8-s time interval. Test stimuli consisted of 14,16, 18, 20 or 22 flashes presented along a 4-s time interval. Flashes were evenly distributed throughout the period, with a random jitter applied in the interval between flashes (±15% of the exact inter-flash distance). This resulted in High numerosity adaptor having a frequency of ~8 Hz, Low numerosity adaptor having a frequency of ~2 Hz and test stimuli having a frequency of ~3.5, ~4, ~4.5, ~5, and ~ 5.5 Hz.

### Experimental paradigm and procedure

**Figure 1:**
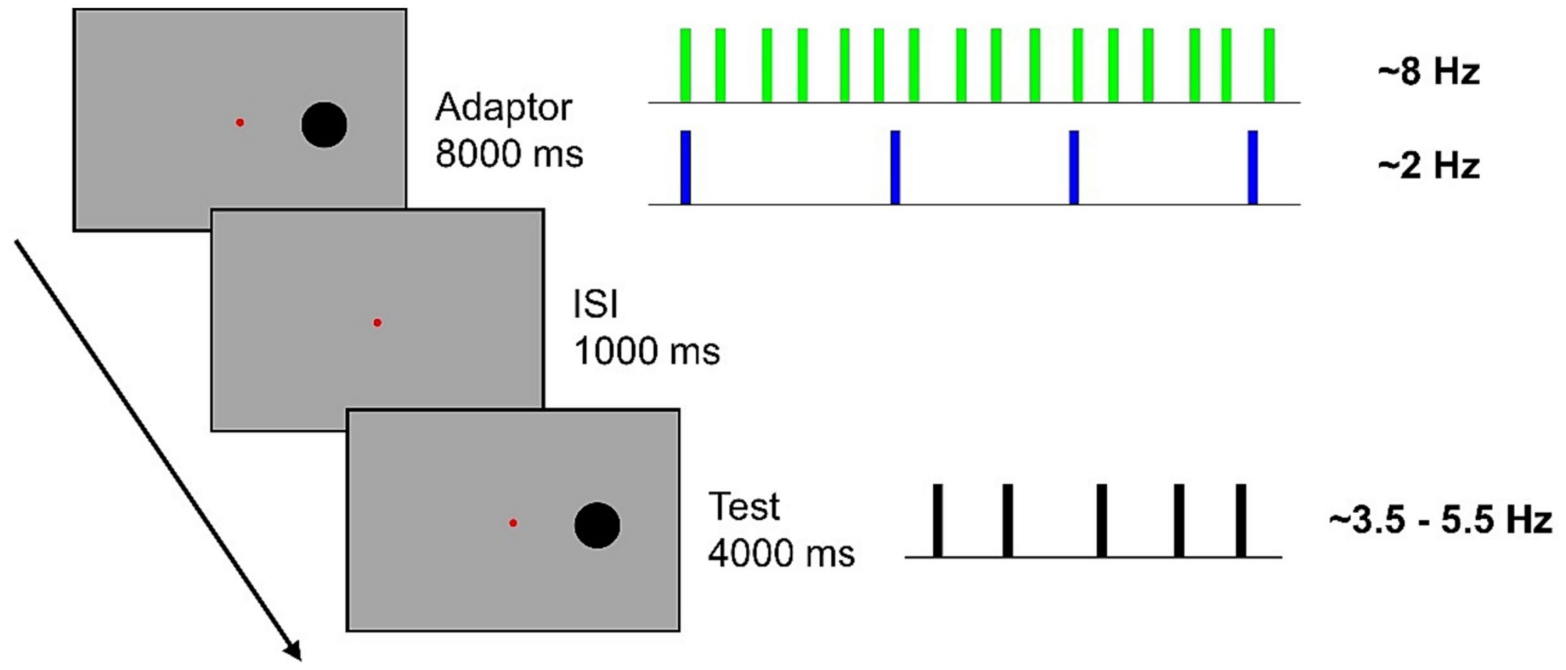
In a blocked design, participants were presented with an 8-s adaptation sequence comprising either 64 flashes (~8 Hz) or 16 flashes (~2 Hz). Subsequently, they were required to estimate the numerosity of a sequence of 14, 16, 18, 20, or 22 flashes presented along a 4-s interval. Flashes were jittered from a perfectly evenly distribution, with a random 15% inter-flash time.

A typical trial began with an adaptation sequence followed by an ISI (1,000 ms) and a test sequence (Figure 1). Participants were asked to verbally report the perceived numerosity of the test sequence and an experimenter, blind to the stimuli, typed answer on the keyboard.

A total of 350 trials divided in 10 blocks of 35 trials each, were presented. For half of the participants the first five blocks contained sequences of High adaptation while for the other half of participants the first five blocks consisted of Low adaptation. During the task, participants were asked to fixate on the central fixation point and to keep as still as possible. To anchor estimation judgments within the correct numerical range, at the beginning of each block participants were familiarized with the presentation of the lowest and the highest numerous test sequence. Each block lasted ~15 min and short inter-blocks brakes were administered.

### EEG recording and preprocessing

EEG signal was recorded with a g. Nautilus Multi-Purpose system (gTEC, Austria) from 30 g.SCARABEO active gel-based electrodes (FP1, FP2, F7, F3, Fz, F4, F8, FC5, FC1, FC2, FC6, C3, Cz, C4, CP5, CP1, CP2, CP6, P7, P3, Pz, P4, P8, PO7, PO3, POz, PO4, PO8, O1, O2) while electrooculogram (EOG) signal was recorded from two electrodes positioned on the outer canthi of both eyes. The signal was referenced online to the right earlobe and the ground electrode was placed on AFz. Impedances were kept below 30 kΩ. The signal was recorded with a high-pass filter of 0.01 Hz and digitized at a sampling rate of 500 Hz.

Pre-processing was carried out using custom routines in MATLAB R2021b (The MathWorks, Inc., Natick, Massachusetts USA) and EEGLAB v2021.1 (Delorme and Makeig, 2004). First, the signal was band-pass filtered from 1 to 40 Hz (type: FIR; cut-off frequency: −6 dB; 0.5 40.5 Hz). Subsequently, bad channels were interpolated (average interpolated channels: 0.87) and signal was offline re-referenced to the average of the cephalic electrodes. Epochs (−500 to 4,500 ms) corresponding to the presentation of the test stimulus were extracted from the continuous EEG and those containing excessive muscular artifacts were discarded by visual inspection (average removed epochs: 7.1%). Infomax independent component analysis (ICA) algorithm was run and components corresponding to vertical eye-movements and to residual anterior muscle artifacts were removed (average removed ICs: 7.5).

### Event-related potentials

To extract event-related potentials (ERPs) to the onset of the test stimulus, epochs were trimmed from −100 to 200 ms and baseline period was removed (−100 to 0). Then, ERP responses elicited by trials corresponding to numerosities 14, 16, 18, and 20 were selected and averaged within each adapting conditions. We excluded numerosity 22 as for this numerosity the appearance of the second flash of the test sequence occurred earlier (~180 ms) than the end of the epoch (200 ms). The rationale for selecting such a short epoch was related to temporal constraint induced by the appearance of the second flash. Picking the first 200 ms of the ERP response allowed us to maintain a good representation of the early visual elaboration (i.e., C1 and P1) while maintaining a sufficient number of trials.

### Steady-state visual evoked potentials

Selective averaging of the epochs was performed separately for each participant and each experimental condition and the spectral magnitude obtained during the 4,000 ms presentation of the test stimulus was computed using fast Fourier transformation (FFT) analysis implemented in the “freqtag_FFT” function of the FreqTag toolbox (Figueira et al., 2022). ssVEP responses were quantified as the power measured at the tagging frequency corresponding to each numerosity. This is because 14, 16, 18, 20, and 22 flashes quasi-periodically displayed along a 4,000 ms time interval correspond approximately to frequency tagging stimulations of 3.5, 4, 4.5, 5, and 5.5 Hz. For each participant, power amplitude was then averaged across numerosities but not across the two adapting conditions.

### Phase-based functional connectivity analysis

Functional connectivity was computed through a measure of phase coherence known as inter-site phase clustering (ISPC). ISPC reflects the degree to which the phase angle differences between a given pair of electrodes are clustered in polar space (Cohen, 2014; Cohen, 2015). Prior to ISPC analysis a surface Laplacian filter was applied to artifact-free EEG data epochs. Surface Laplacian is a spatial bandpass filter that attenuates low spatial frequencies and helps to minimize spurious connectivity effects arising from volume conductance (Perrin et al., 1989; Cohen, 2015). Following the application of Surface Laplacian, trials were averaged within adapting conditions (High and Low) and within numerosity (14, 16, 18, 20, and 22). Then EEG signal was Hilbert transformed and filtered at the very specific tagging frequency of each numerosity (i.e., 3.5, 4, 4.5, 5, and 5.5 Hz) using a second order Butterworth filter implemented in the “freqtag_HILB” function of the FreqTag toolbox (Figueira et al., 2022). ISPC was then evaluated separately for all pairs of electrodes, all participants and all tagging frequencies. The resulting connectivity matrices were then averaged across numerosities but not across the two adapting conditions.

### Statistical analyses

Behavioral data were analyzed using a 2 × 5 repeated measures ANOVA with the within factors Adaptation (Low, High) and Numerosity (14, 16, 18, 20, 22). To compensate for violations of sphericity, Greenhouse–Geisser corrections were applied (Greenhouse and Geisser, 1959) and corrected *p*-values (but uncorrected degrees of freedom) are reported. Post-hoc comparisons were performed using the Bonferroni correction.

Electrophysiological data were analyzed through non-parametric permutation tests using 15.000 permutations as implemented in EEGLAB function “statcond” (Delorme and Makeig, 2004). Benjamini-Hochberg false discovery rate (FDR) correction was used to adjust *p*-values for multiple comparisons (Benjamini and Hochberg, 1995).

## Results

### Behavioral

**Figure 2:**
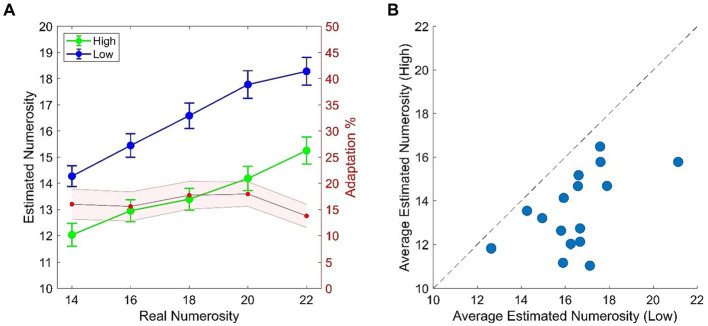
**(A)** Behavioral results from the temporal numerosity adaptation task. Plots depicts numerical estimates averaged across participants for the High (Green) and the Low (Blue) adapting conditions. Bars represent SEM. Red shaded plot represent the percentage of adaptation (i.e., estimated Low – estimated High/real) across tested numerical ranges. Shaded area represents SEM. **(B)** Scatterplot of average numerical estimates in High adapting condition against the Low adapting condition. Dots represent single participant’s data.

In light of the typical temporal numerosity adaptation effect (e.g., Arrighi et al., 2014; Grasso et al., 2022a) we expected a consistent difference in the numerical estimation of the test when this was preceded by the High (~8 Hz) or the Low (~2 Hz) adaptor. More specifically, reduced numerosity estimates are expected in the High adaptation condition as compared to the Low adaptation. This expectation was confirmed by empirical data which revealed that numerosity estimates in the High adaptation condition were reduced by ~15% as compared to the Low adaptation condition with this result being consistent across all numerical ranges (Figure 2A). All participants got adapted, although to a variable extent (Figure 2B), as also confirmed by the two-way ANOVA which showed a highly significant main effect of Adaptation [*F*_(1, 15)_ = 45.818; *p* < 0.001; *ƞ_p_^2^* = 0.75] and Adaptation x Numerosity [*F*_(4, 60)_ = 9.667; *p =* 0.001; *ƞ_p_^2^* = 0.39]. Adaptation occurred across all test numerosities (all comparisons of interest had *ps* < 0.001) and, as expected, also a main effect of Numerosity emerged [*F*_(4, 60)_ = 116.111; *p* < 0.001; *ƞ_p_^2^* = 0.88].

### Event related potentials

**Figure 3:**
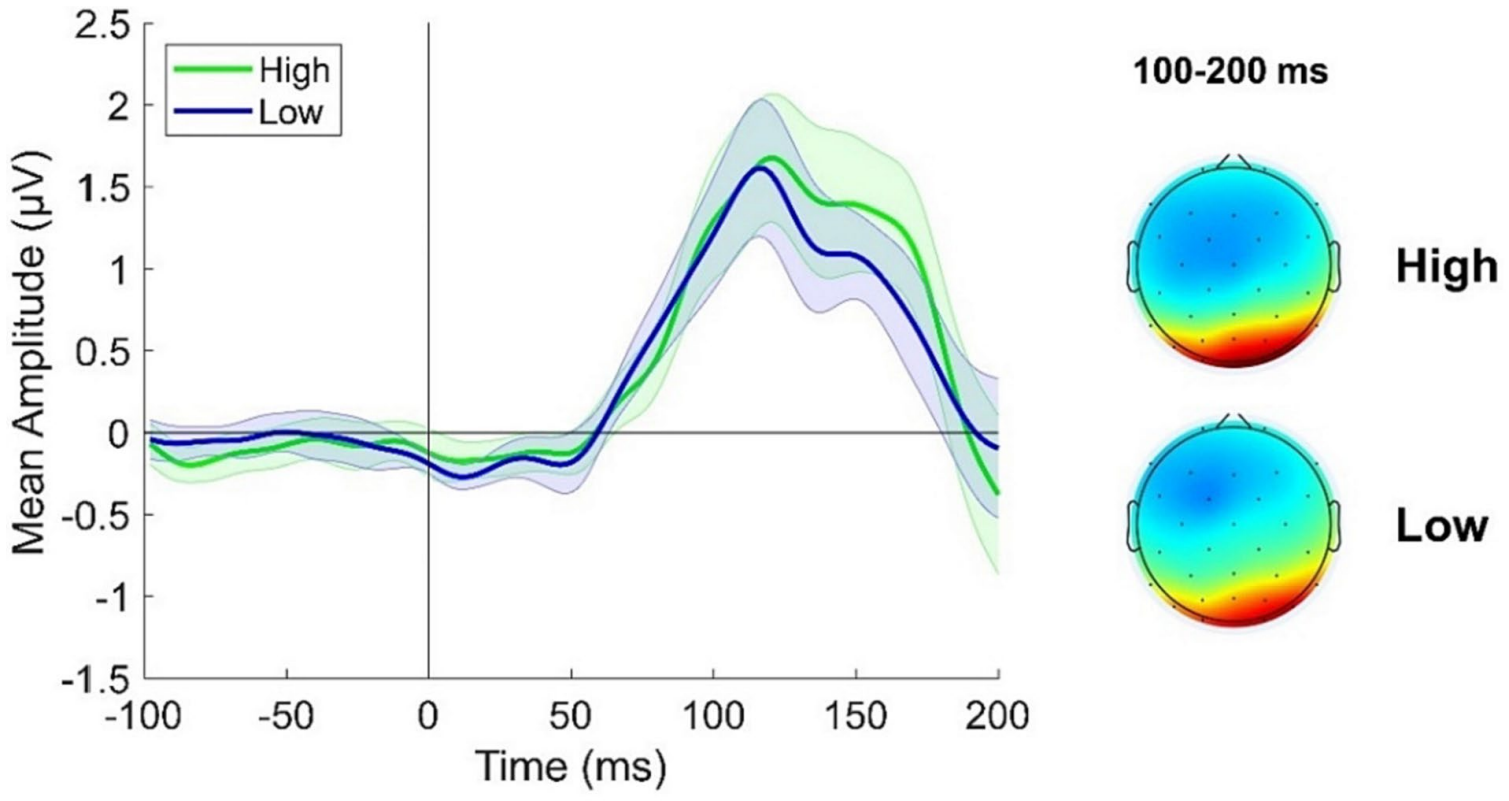
ERPs response averaged across electrodes PO3, PO4, POz, PO7, PO8, O1, O2 for the High adapting condition (green ERP) and the Low adapting condition (blue ERP). Topographies depict the spatial distribution of ERP response in the two conditions averaged between 100 and 200 ms. Shaded areas represent SEM.

A cluster of posterior electrodes was *a priori* chosen as we here inspected components occurring before 200 ms from stimulus onset which are known to reflect activity from relatively early visual areas. ERPs were derived from the average response recorded over electrodes PO3, PO4, POz, PO7, PO8, O1 and O2. Looking at Figure 3 it is evident a slight increase in ERP response in the High adapting condition as compared to Low adapting around 100 and 200 ms post stimulus onset. However, this difference did not reach statistical significance (all *ps* > 0.65) suggesting that at this very early stage of test stimulus processing no relevant influence of previous adaptation sequence emerge.

### Steady-state visual evoked potentials

**Figure 4:**
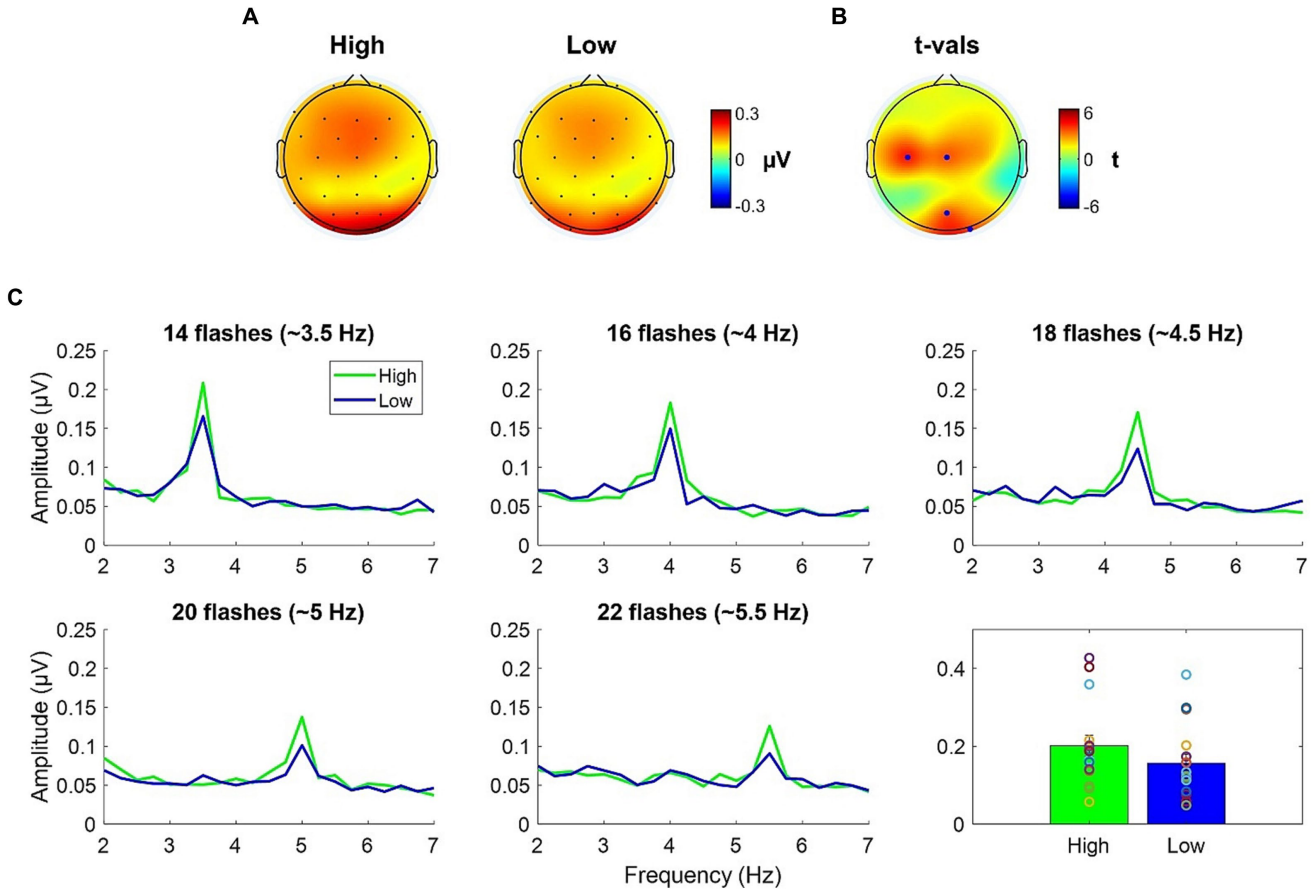
**(A)** ssVEP topographies of the averaged across-numerosity power amplitude in the High and the Low adapting conditions. **(B)** *t*-values topography of the comparison between High and Low conditions; blue dots indicate the electrodes where the power amplitude in the High condition was significantly higher than in the Low condition after FDR correction. **(C)** Spectral magnitude as a function of frequency for each numerical range (14 to 22) and each adapting condition (High and Low). Bottom right plot depicts the power amplitude averaged across numerosities.

**Figure 5:**
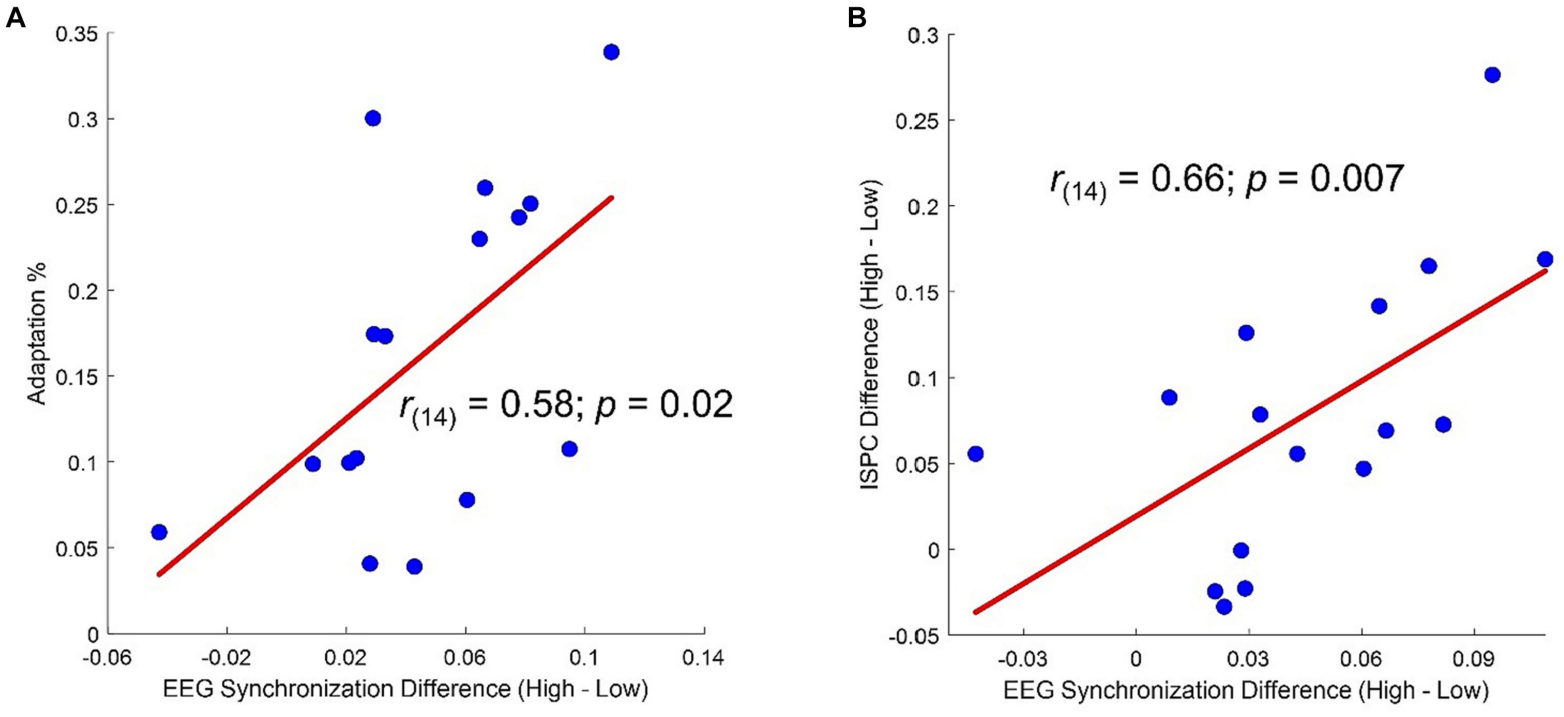
**(A)** Correlation between percentage of adaptation (i.e., estimated Low – estimated High/real) and difference in average ssVEP amplitude (High – Low). **(B)** Correlation between difference in average ssVEP amplitude (High – Low) and difference in average ISPC (High – Low).

Topographies of the averages across-numerosity power amplitude in the High and Low adapting conditions are shown in Figure 4A. For the analysis of steady state visual evoked potentials (ssVEP), we did not *a priori* choose a specific cluster of electrodes as the repeated visual stimulation at a given frequency is known to produce changes within large scale cortical networks (Zhang et al., 2013). Permutation test revealed a significant difference between ssVEP elicited by the tagging frequency of the test stimulus when participants were previously exposed to the High adaptor as compared to when participants were exposed to the Low adaptor. This difference was evident across four sensors (all *ps* < 0.03), two of which were distributed occipitally (i.e., POz and O2) while the other two were distributed centrally (i.e., C3 and Cz; see Figure 4B). ssVEP showed that, although the test stimulus had a quasi-periodical flashing, the brain was perfectly synchronized within the prevailing stimulation frequency and the amplitude of the power measured at the tagging frequency was consistently larger in the High adaptation condition at all the tested numerosities (Figure 4C). Moreover, to confirm that this variation was related to changes in behavioral performances we correlated individual differences in average ssVEP obtained subtracting High from Low power amplitudes with individual differences in the percentage of numerosity adaptation (i.e., Low – High). A significant positive relationship emerged between the two measures [Spearman; *r*_(14)_ = 0.58; *p* = 0.02]. The larger was the amount of adaptation, the larger was the absolute difference in the EEG synchronization between the two adapting conditions (Figure 5A).

### Inter-site phase clustering

**Figure 6:**
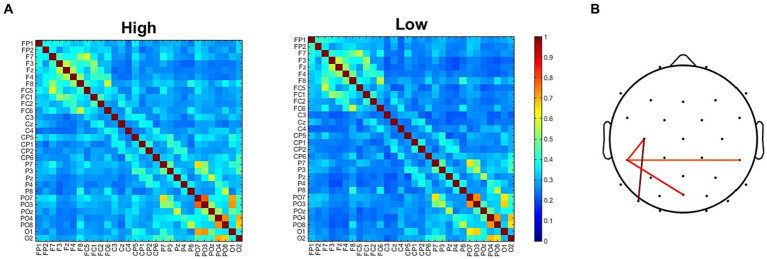
**(A)** ISPC connectivity matrices for the High and the Low adapting conditions. **(B)** Electrodes pairs showing larger ISPC values in the High adapting condition.

To further investigate the neural underpinnings of the modifications induced by the two adapting conditions we analyzed functional connectivity. The analysis of ssVEP clearly revealed two clusters of electrodes showing the maximal difference in synchronization between the two adapting conditions. It remained to be understood whether this difference was related to changes in interareal communications. For this reason, we compared ISPC matrix obtained in the High adaptation condition with that obtained in the Low adaptation condition (Figure 6A). Results revealed four electrode pairs (C3-CP5, CP5-CP6, CP5-Poz, C3-PO7) showing larger ISPC in the High adaptation condition (all *ps* < 0.05, FDR corrected). Importantly these connections comprised two out of four of the electrodes that also showed larger ssVEP differences (Figure 6B).

Moreover, a correlation analysis revealed that participants showing the larger differences in ISPC between High and Low adaptation conditions within the selected pairs of electrodes also had the largest difference in ssVEP between High and Low adaptation conditions (Figure 5B).

## Discussion

In the present study we investigated the neural underpinnings of temporal numerosity adaptation. We used a classical adaptation paradigm to unveil changes in neural responses to a test stimulus sequence when this was preceded by a high or low numerous adapting sequence. First of all, behavioral results replicated previous findings by showing the expected numerosity adaptation effect which led to an underestimation of the test sequence when this was preceded by a high numerous sequence as compared to when it was preceded by a low numerous sequence. We here showed that this behavior correlated with the amplitude of the frequency tagging response induced by the test stimulus. In line with previous literature, our results revealed that ssVEP elicited by a sequential stimulation is robustly influenced by the state of the system (for a review see Norcia et al., 2015). ssVEP response to test stimulus sequence was less robust when participants were previously exposed to a Low adapting sequence as compared to when exposed to a High adapting sequence. Crucially, this pattern was tightly linked with behavioral performances. The larger was the amplitude difference between ssVEP in the High and Low adapting conditions the larger was the percentage of numerical adaptation experienced by participants. This result complements previous findings within the spatial domain where we showed that a mid-latency posteriorly distributed component of the ERP response is capable to track the amount of perceptual bias induced by spatial numerosity adaptation (Grasso et al., 2022b), a result in line with previous fMRI evidence reporting changes in numerosity preferences of numerosity-tuned neural populations (Piazza et al., 2004, 2007; Tsouli et al., 2020, 2021). Here we showed that the exposure to a highly numerous adapting sequence increases cortical response to test stimulus producing a more vigorous synchronization. Although one could expect an enhanced electrophysiological response to be associated with more proficient behaviors, it is well documented that this is not always the case as an increase in cortical excitability has been often associated with detrimental performances at the behavioral level (Grasso et al., 2020c, 2021c). We showed a similar trend with increased cortical responses to the test stimulus being associated with larger numerical estimation biases of the test sequence, suggesting that a high frequency repetitive visual stimulation leads to a detrimental increase in cortical excitability which may prevent the system from an optimal parceling of subsequent visual information. Put in another way, underestimation behaviors in a temporal numerosity estimation task might be associated with increased cortical excitability mediated by previous exposure to a high frequency adaptor. Interestingly, our results also showed that this was evident across two distinct clusters of electrodes covering both posterior and anterior sites suggesting that the adaptor-mediated modulation of cortical excitability was not confined to early visual areas. Congruently, functional connectivity analysis highlighted an enhanced antero-posterior connectivity in the High adaptation condition which likely mirrors ssVEP results as also suggested by the significant correlation found between the two measures. We speculate that such enhanced communication could reflect an attempt to compensate the reduced parceling of temporal sequences produced by previous exposure to the High adaptor. This interpretation is derived by studies reporting increased phase-based functional connectivity during the execution of complex tasks or in the elderly (Wang et al., 2018). Nevertheless, we acknowledge that such interpretation is speculative and deserves a more in-depth testing. In addition, ERP responses did not exhibit a significant difference between the two adapting conditions suggesting the idea that temporal numerosity adaptation does not depend on a modified early visuo-cortical response to a generic visual input (i.e., a single flash) but rather reflects a distributed modification of neural responses implicated in the extraction of numerical information from repetitive signals. Importantly, given that such pattern of results was retrieved within the time of test sequence presentation (i.e., before a numerical estimate was even issued), it is likely to reflect a process linked to the encoding of numerical information but not selectively implicated in such operation. We speculate that what we showed here may be reminiscent of the interplay between monotonic responses from early sensory signals and timing-tuned responses from higher levels associative cortices (Zhou et al., 2018; Hendrikx et al., 2022). Timing-dependent signals from early sensory areas are, indeed, transformed into tuned responses to numerosity within higher level cortical areas and temporal numerosity adaptation may act by altering early monotonic responses from which timing-tuned responses are derived (Piazza and Izard, 2009; Tsouli et al., 2021; Paul et al., 2022). In this view, brain’s processing of temporal numerosity may be determined through the processing of other features, such as rate and/or temporal frequency, but still being closely associated with such mechanisms as demonstrated by the strong relationship retrieved in our data.

Importantly, although both current results and those obtained in the spatial domain (Grasso et al., 2022b) highlight a clear modification of cortical response to test stimulus as a marker of numerical adaptation, a few clarifications need to be made. First, while we here showed that the amount of perceptual adaptation is significantly correlated with an increase in adaptor-dependent cortical excitability, this was not the case for spatial numerosity adaptation where adaptation magnitude correlated with the amount of reduction in cortical response to the test stimulus. Second, while the effects produced by spatial numerosity adaptation were retrievable within a relatively early processing time (~200 to 250 ms), temporal numerosity adaptation did not produce any significant difference in the very early response to the test sequence. Finally, scalp topographies of spatial numerosity adaptation revealed a single posteriorly distributed cluster of electrodes exhibiting the largest effect of adaptation, while scalp topographies of temporal numerosity adaptation highlighted the involvement of two distinct clusters of electrodes with an antero-posterior distribution. Taken together, these differences corroborate the idea that, likewise perception, temporal and spatial numerosity adaptation could pertain independent processes being subserved by distinct neural networks (Cavdaroglu and Knops, 2019). Nevertheless, it remains to be understood why spatial and temporal numerosity adaptation were found to interact. For example, it has been reported that there is no measurable cost in reaction times in making cross-format judgments (Barth et al., 2003). Furthermore, Arrighi and collaborators reported that high frequency sequential flashes were capable to produce an underestimation of the numerosity of an array of dots presented around the adaptation area (Arrighi et al., 2014). However, adaptation to the numerosity of the elements in a cloud of dots failed to affect numerosity estimates of sequences of impulses to suggest, at best, only a partial interaction within the two numerical systems. It might be the case that despite being encoded by independent brain circuits located in different areas, at a later stage temporal and spatial numerical information get integrated together. This interpretation is corroborated by a study on primates revealing the existence of segregate populations of neurons in the intraparietal sulcus selectively coding spatial and temporal numerical information successively integrated into a cardinal format-free representation (Nieder et al., 2006).

Finally, despite the participants of the present study were required to accomplish a numerical task, the sequences of visual flashes of variable numerosity we used were also highly distinguishable in terms of temporal frequency. This feature has been reported to be, alike numerosity, susceptible of adaptation (Levitan et al., 2015) so it might be possible to interpret the present results also in terms of adaptation to perceived rate. In this view, differences in inter-flash intervals between the two adapting sequences may have had an impact on our results. Importantly, the relationship between temporal numerosity and frequency has been recently investigated in study where participants had to reproduce a sequence of visual impulses without being instructed about the visual feature to leverage on to accomplish the task (Cicchini et al., 2023). The results indicates that the visual feature the participants primarily relied on to reproduce the visual sequence was its numerosity but a significant weight was also found for the temporal frequency to suggest that both dimensions are salient for the encoding of sequence of visual impulses. However, as previous studies reported that temporal frequency is strongly processed by an hierarchy of visual areas including both sub-cortical and early cortical circuits (D’Souza et al., 2011), in the case the adaptation signatures reported in this study tagged temporal frequency and not numerosity, we might have expected to find a signature of adaptation at an early stage alike it has been reported to be the case for spatial numerosity. On the contrary, as reported above, no significant difference was found among the experimental conditions in the very early response to suggest that the encoding of a “late” component was that triggering the ERP signal amplitude. Obviously, no definitive conclusion can be drawn in light of the present data so only future studies designed to finely manipulate the stimuli properties to put at odds numerical and temporal frequency (i.e., by varying the length of the test sequence) will be able to resolve this issue. Finally, we here employed a high adaptor sequence tapping the low boundary of alpha rhythm, an oscillation range inherently linked to the processing of visual information and distractor suppression (e.g., Pfurtscheller, 2003; Thut et al., 2006; Klimesch et al., 2011; Grasso et al., 2020b). In this view, although the increase in cortical excitability to test stimulus may result from an increased adaptor-mediated alpha synchronization, the correlation with changes in numerical estimates suggests a genuine association with the encoding of numerical information. Accordingly, previous studies showed that underestimation of numerical sequences can be obtained also when the adaptation period is characterized by motor sequences lying within the theta range (Anobile et al., 2016; Burr et al., 2019).

To conclude, the present study not only highlight the ability of the brain to shape its responses based on varying external conditions (for a recent review on visual plasticity see Grasso et al., 2020a) but also that such modifications are likely outreached through the recruitment of large scale cortical networks. We here showed that visual plastic phenomena related to the encoding of temporal numerosity information reflect changes in rhythmic evoked activity that are likely related to long range communications between distinct brain regions. This result also supports the notion that spatial and temporal numerical estimates reflect the activity of segregated neural mechanisms.

## Data availability statement

The raw behavioral and electrophysiological data have been deposited at Zenodo repository and are publicly available at the following address: https://doi.org/10.5281/zenodo.10696430.

## Ethics statement

The studies involving humans were approved by “Commissione per l’Etica della Ricerca,” University of Florence, 23rd September 2021, n. 174. The studies were conducted in accordance with the local legislation and institutional requirements. The participants provided their written informed consent to participate in this study.

## Author contributions

PG: Conceptualization, Data curation, Formal analysis, Investigation, Methodology, Project administration, Software, Writing – original draft, Writing – review & editing. IP: Data curation, Project administration, Writing – original draft. FC: Data curation, Writing – review & editing. RA: Funding acquisition, Resources, Supervision, Writing – review & editing.
